# Allergen immunotherapy for insect venom allergy: protocol for a systematic review

**DOI:** 10.1186/s13601-016-0095-x

**Published:** 2016-02-16

**Authors:** Sangeeta Dhami, Ulugbek Nurmatov, Eva-Maria Varga, Gunter Sturm, Antonella Muraro, Cezmi A. Akdis, Darío Antolín-Amérigo, M. Beatrice Bilò, Danijela Bokanovic, Moises A. Calderon, Ewa Cichocka-Jarosz, Joanna N. G. Oude Elberink, Radoslaw Gawlik, Thilo Jakob, Mitja Kosnik, Joanna Lange, Ervin Mingomataj, Dimitris I. Mitsias, Holger Mosbech, Oliver Pfaar, Constantinos Pitsios, Valerio Pravettoni, Graham Roberts, Franziska Ruëff, Betül Ayşe Sin, Aziz Sheikh

**Affiliations:** 1 Evidence-Based Health Care Ltd, Edinburgh, UK; 2 Systematic Review at Decision Resources Group, Abacus International, Bicester, UK; 3 Respiratory and Allergic Disease Division, Department of Pediatric and Adolescent Medicine, Medical University of Graz, Graz, Austria; 4 Department of Dermatology and Venerology, Medical University of Graz and Outpatient Allergy Clinic Reumannplatz, Vienna, Austria; 5 Department of Women and Child Health, Food Allergy Referral Centre Veneto Region, Padua General University Hospital, Padua, Italy; 6 Swiss Institute of Allergy and Asthma Research (SIAF), University of Zurich, Zurich, Switzerland; 7 Servicio de Enfermedades del Sistema Inmune-Alergia, Departamento de Medicina y Especialidades Médicas, Hospital Universitario Príncipe de Asturias, Madrid, Spain; 8 Universidad de Alcalá, Madrid, Spain; 9 Allergy Unit, Department of Internal Medicine, University Hospital of Ancona, Ancona, Italy; 10 Department of Dermatology and Venerology, Medical University of Graz, Graz, Austria; 11 Section of Allergy and Clinical Immunology, Imperial College London, London, UK; 12 National Heart and Lung Institute, Royal Brompton Hospital, London, UK; 13 Department of Pediatrics, Jagiellonian University Medical College, Kraków, Poland; 14 Department of Allergology Internal Medicine, University Medical Hospital Groningen, University of Groningen, Groningen, The Netherlands; 15 Department of Internal Medicine, Allergy and Clinical Immunology, Medical University of Silesia, Katowice, Poland; 16 Department of Dermatology and Allergology, University Medical Center Gießen and Marburg (UKGM), Justus Liebig University Gießen, Gießen, Germany; 17 Medical Faculty Ljubljana, University Clinic of Respiratory and Allergic Diseases Golnik, Golnik, Slovenia; 18 Department of Pediatric Pneumonology and Allergy, Medical University of Warsaw, Warsaw, Poland; 19 Department of Allergollogy and Clinical Immunology, Mother Theresa School of Medicine, Tirana, Albania; 20 Department of Paraclinical Disciplines, Faculty of Technical-Medical Sciences, Medicine University of Tirana, Tirana, Albania; 21 Department of Allergy and Clinical Immunology, 2nd Pediatric Clinic, University of Athens, Athens, Greece; 22 Allergy Clinic, Copenhagen University Hospital Gentofte, Gentofte, Denmark; 23 Department of Otorhinolaryngology, Head and Neck Surgery, Center for Rhinology and Allergology, University Hospital, Mannheim, Wiesbaden, Germany; 24 Department of Nutrition and Dietetics, Harokopio University, Athens, Greece; 25 Allergy Outpatient Clinic, Apollonio Hospital, Nicosia, Cyprus; 26 UOC Clinical Allergy and Immunology, IRCCS Foundation Ca’ Granda Ospedale Maggiore Policlinico, Milan, Italy; 27 NIHR Respiratory Biomedical Research Unit, The David Hide Asthma and Allergy Research Centre, St Mary’s Hospital, Newport Isle of Wight, University Hospital Southampton NHS Foundation Trust, Southampton, UK; 28 Faculty of Medicine, University of Southampton, Southampton, UK; 29 Klinik und Poliklinik für Dermatologie und Allergologie, Ludwig-Maximilians-Universität, Munich, Germany; 30 Division of Immunology and Allergy, Department of Pulmonary Diseases, Faculty of Medicine, Ankara University, Ankara, Turkey; 31 Allergy and Respiratory Research Group, The University of Edinburgh, Edinburgh, UK

**Keywords:** Allergy, Insect sting, Hymenoptera venom allergy, Insect venom allergy, Allergen immunotherapy, Systemic sting reaction

## Abstract

**Background:**

The European Academy of Allergy and Clinical Immunology (EAACI) is in the process of developing the EAACI Guidelines for Allergen Immunotherapy (AIT) for the Management of Insect Venom Allergy. We seek to critically assess the effectiveness, cost-effectiveness and safety of AIT in the management of insect venom allergy.

**Methods:**

We will undertake a systematic review, which will involve searching international biomedical databases for published, in progress and unpublished evidence. Studies will be independently screened against pre-defined eligibility criteria and critically appraised using established instruments. Data will be descriptively and, if possible and appropriate, quantitatively synthesised.

**Discussion:**

The findings from this review will be used to inform the development of recomendations for EAACI’s Guidelines on AIT.

## Background

Hymenoptera venom allergy is a potentially life-threatening allergic reaction following one or more stings to bees, wasps, polistes, hornets or fire ants. The risk of anaphylaxis to hymenoptera stings is greater in adults when compared to children due to increased sting exposure, co-morbidities and concomitant medications in this age group. Systemic reactions have been reported in up to 3 % of adults and 0.34 % of children [1, 2].

Symptoms range from large local reactions at the sting site to mild, moderate, and severe systemic reactions. Mild systemic reactions are usually generalized skin symptoms such as flush, urticaria and angioedema. Typically, dizziness, dyspnea, nausea are moderate symptoms while anaphylactic shock, asthma, loss of consciousness, or even cardiac or respiratory arrest all indicate a severe sting reaction. The fear of future reactions usually greatly impairs quality of life. Around a quarter of fatalities from anaphylaxis are triggered by venom allergy [3–5].

Patients are advised to carry an emergency kit containing H1-antihistamines, corticosteroids, and adrenaline (epinephrine) depending on their previous sting reaction. The only treatment that can potentially prevent further severe reactions is venom immunotherapy (VIT). This may be effective with long-term clinical benefit and improved quality of life [6, 7]. However, despite its life-saving potential, VIT is still being under-prescribed and under-used in Europe [8].

The European Academy of Allergy and Clinical Immunology (EAACI) is in the process of developing the EAACI Guidelines for AIT. This systematic review is one of five inter-linked evidence syntheses that are being undertaken in order to provide a state-of-the-art synopsis of the current evidence base in relation to evaluating AIT for the treatment of insect venom allergy, allergic rhinoconjunctivitis, food allergy and allergic asthma, and allergy prevention. These will be used to inform the formulation of key clinical recommendations for subsequent clinical guidelines. The focus of this review is on assessing the effectiveness, safety and cost-effectiveness of VIT in the treatment of insect venom allergy.

## Methods

### Search strategy

**Fig. 1:**
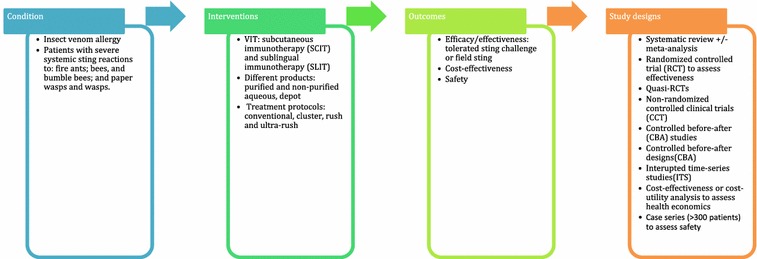
Conceptualization of systematic review of allergen immunotherapy for insect venom allergy

A highly sensitive search strategy has been developed, and validated study design filters will be applied to retrieve all articles pertaining to the use of VIT for insect venom allergy from electronic bibliographic databases. We have conceptualized the search to incorporate the four elements below as shown in Fig. 1.

To retrieve systematic reviews, we will use the systematic review filter developed at McMaster University Health Information Research Unit (HIRU) (http://hiru.mcmaster.ca/hiru/HIRU_Hedges_MEDLINE_Strategies.aspx#Reviews). To retrieve randomized controlled trials (RCTs), we will apply the Cochrane highly sensitive search strategy for identifying RCTs in MEDLINE [9]. To retrieve non-randomized studies, i.e. controlled clinical trials (CCTs), controlled before-and-after (CBA) and interrupted time-series (ITS) studies, we will use the Cochrane Effective Practice and Organisation of Care (EPOC) filter Version 2.4, available on request from the EPOC Group [10, 11]. To retrieve case series, we will use the filter developed by librarians at Clinical Evidence: http://clinicalevidence.bmj.com/x/set/static/ebm/learn/665076.html.

We will search the following databases:

- Cochrane Library including:
  - Cochrane Database of Systematic Reviews (CDSR)
  - Database of Reviews of Effectiveness (DARE)
  - CENTRAL (Trials)
  - Methods Studies
  - Health Technology Assessments (HTA)
  - Economic Evaluations Database (EED)
- MEDLINE (OVID)
- Embase (OVID)
- CINAHL (Ebscohost)
- ISI Web of Science (Thomson Web of Knowledge)
- TRIP Database (www.tripdatabase.com)
- Clinicaltrials.gov (NIH web).
- Clinicaltrialsregister.eu
- Current controlled trials (www.controlled-trials.com)
- Australian and New Zealand Clinical Trials Registry (http://www.anzctr.org.au).

The search strategy has been developed on OVID MEDLINE and then adapted for the other databases (see “Appendix”). In all cases, the databases will be searched from inception to October 31, 2015. Additional references will be located through searching the references cited by the identified studies, and unpublished work and research in progress will be identified through discussion with experts in the field. We will invite experts who are active in the field from a range of disciplines and regions to add to the list of included studies by identifying additional published and unpublished papers they are aware of and research in progress. There will be no language restrictions employed; where possible, all relevant literature will be translated into English.

### Inclusion criteria

#### Patient characteristics

We are interested in identifying studies conducted on patients of any age with a physician confirmed diagnosis of systemic sting reaction to a venom sting from fire ants, bees and bumble bees, and paper wasps and wasps.

#### Interventions of interest

This review is focused on VIT using different products (purified and non-purified aqueous or depot) and different treatment protocols (conventional, cluster, rush and ultra-rush) adminsitered through the SCIT or SLIT routes.

#### Comparators

We are interested in studies comparing VIT with placebo or no treatment (i.e. a natural course of the disease).

#### Study designs

Systematic reviews of RCTs, and RCTs, will be used to investigate effectiveness; health economic analysis will be used to assess cost-effectiveness; and systematic reviews, and RCTs and case series with a minimum of 300 patients will be used to assess safety. We will appraise the evidence by looking at higher levels of evidence such as systematic reviews and/or meta-analyses of RCTs, together with individual RCTs. However, given the likelihood that we will find only a limited number of RCTs, we will also search for and include quasi-RCTs—[i.e. non-randomized CCTs, controlled before and after (CBA) studies and interrupted time series (ITS) analyses]. Given the high inherent risk of bias in making inferences from quasi-RCTs, clinical recommendations will be based on the findings from randomized controlled trials and the quasi-randomized controlled trials will only be used to guide suggestions on which areas need to be prioritized in future research [12].

#### Outcomes

***Primary***

- Efficacy assessed by tolerated sting challenge or field sting both short-term and long-term, where long-term is defined as sustained clinical efficacy after discontinuation of treatment (VIT).

***Secondary***

- Assessment of disease specific quality of life;
- Safety as assessed by local and systemic reactions in accordance with the World Allergy Organization’s grading system of side-effects [13, 14];
- Health economic analysis from the perspective of the health system/payer.

### Exclusion criteria

- Reviews, discussion papers, non-research letters and editorials;
- Animal studies;
- Quantitative studies not employing systematic review or RCT or quasi-RCT designs;
- Qualitative studies;
- Case series (<300 patients).

### Study selection

All references will be uploaded into the systematic review software Distiller and undergo initial de-duplication. Study titles will be independently checked by two reviewers according to the above selection criteria and categorized as: included, not included or unsure. For those papers in the unsure category, we will retrieve the abstract and re-categorize as above. Any discrepancies will be resolved through discussion and, if necessary, a third reviewer will be consulted. Full text copies of potentially relevant studies will be obtained and their eligibility for inclusion independently assessed. Studies that do not fulfil all of the inclusion criteria will be excluded.

### Quality assessment strategy

Quality assessments will independently be carried out on each study by two reviewers using the relevant version of the Critical Appraisal Skills Programme (CASP) quality assessment tool for systematic reviews and health economic evaluations [15]. We will assess the risk of bias of experimental studies using the criteria suggested by the Cochrane EPOC Group [16]. RCTs, CCTs and CBAs will be assessed for generation of allocation sequence, concealment of allocation, baseline outcome measurements, baseline characteristics, incomplete outcome data, blinding of outcome assessor, protection against contamination, selective outcome reporting and other risks of bias using the Cochrane risk of bias tool. For ITS designs, we will also assess the independence of the intervention from secular trends, the pre-specified shape of the intervention and if the intervention may have had an impact on data collection. These methodological assessments will draw on the principles incorporated into the Cochrane EPOC guidelines for assessing intervention studies [17]. Similarly, we will use the quality assessment form produced by the National Institute for Health and Clinical Excellence (NICE) to critically appraise case series [18]. Any discrepancies will be resolved by discussion or, if agreement could not be reached, by arbitration by a third reviewer.

### Analysis, data synthesis and reporting

Data will be independently extracted onto a customized data extraction sheet in Distiller by two reviewers, and any discrepancies will be resolved by discussion or, if agreement cannot be reached, by arbitration by a third reviewer.

A descriptive summary with data tables will be produced to summarize the literature. If clinically and statistically appropriate, meta-analysis using either fixed-effect or random-effects modeling will be undertaken [9]. A narrative synthesis of the data will also be undertaken.

### Sensitivity and subgroup analyses, and assessment for publication bias

Sensitivity analyses will be undertaken by comparing the summary estimates obtained by excluding studies judged to be at high risk of bias.

Subgroup analyses will be undertaken to compare:

- Children (5–11 years) versus adolescents (12–17 years) versus adults (≥18 years);
- Conventional versus cluster versus rush versus ultra-rush protocols in SCIT;
- Conventional in SLIT versus SCIT;
- 3 versus 5 years of treatment;
- Different allergen doses (50 vs. 100 vs. 200 µg of maintenance VIT);
- Bee versus wasp venom;
- Comparing outcomes between those with and without co-existent mast cell disease [19].

Where possible, publication bias will be assessed through the creation of funnel plots, and tested by Egger’s regression test and Begg’s rank correlation test [20, 21].

### Registration and reporting

This review will be registered with the International Prospective Register of Systematic Reviews (PROSPERO): http://www.crd.york.ac.uk/prospero/. The Preferred Reporting Items for Systematic Reviews and Meta-Analyses (PRISMA) checklist will be used to guide the reporting of the systematic review: http://www.prisma-statement.org/.

## Discussion

This review will involve systematically identifying, critiquing and synthesizing the evidence on the efficacy/effectiveness, cost-effectiveness and safety of VIT for the management of venom allergy. The findings from this review will be used to inform the development of recommendations for EAACI’s Guidelines on AIT. We anticipate that this review will be reported in 2016.

## Appendix: Search strategy

### Search strategy 1 (MEDLINE, EMBASE)

1. insect sting.mp. or exp insect sting/
2. insect bite.mp. or exp insect bite/
3. insect allergy.mp. or exp insect allergy/
4. exp immediate type hypersensitivity/or exp delayed hypersensitivity/or exp hypersensitivity/or hypersensitivity.mp.
5. hypersensitivity reaction.mp. or allergic reaction/
6. anaphyla$.mp.
7. systemic anaphylaxis/or exp anaphylaxis/or anaphylaxis.mp.
8. systemic reaction.mp.
9. shock.mp. or anaphylactic shock/or exp traumatic shock/or exp shock/
10. hives.mp. or exp urticaria/
11. laryngeal obstruction.mp. or exp larynx stenosis/
12. angioedema.mp.
13. airway obstruction.mp. or exp airway obstruction/
14. exp Hymenoptera venom/or exp Hymenoptera/or hymenoptera.mp.
15. exp bee venom/or exp bee/or bee.mp.
16. honey bee.mp. or exp honeybee/
17. wasp*.mp. or wasp venom.mp. or exp wasp venom/
18. exp ant sting/or ant.mp. or exp ant/or exp ant venom/
19. sawfl*.mp.
20. (apis mellifera or vespid or vespula or white hornet or yellow jacket or yellow hornet or polistes or arthropod venom or solenopsis invicta or myrmecia pilosula).mp.
21. or/1–20
22. immunotherapy.mp. or exp subcutaneous immunotherapy/or exp immunotherapy/
23. venom immunotherapy.mp.
24. allergen immunotherapy.mp.
25. specific immunotherapy for hymenoptera venom.mp.
26. immunomodulation.mp. or exp immunomodulation/
27. immunologic response.mp. or exp immune response/
28. subcutaneous immunotherapy.mp. or exp subcutaneous immunotherapy/or sublingual immunotherapy.mp. or exp sublingual immunotherapy/
29. (intradermal immunotherapy or intralymphatic immunotherapy).mp.
30. specific immunotherapy.mp.
31. exp systematic desensitization/or exp desensitization/or desensitization.mp.
32. hyposensitization.mp.
33. or/22–32
34. intervention stud*.mp. or exp intervention study/
35. experimental stud*.mp.
36. exp “clinical trial (topic)”/or exp “controlled clinical trial (topic)”/or exp “randomized controlled trial (topic)”/or trial.mp. or exp controlled clinical trial/
37. (randomi?ed controlled trial or non-randomi?ed trial or quasi-randomi?ed trial).mp.
38. placebos.mp. or exp placebo/
39. random allocation.mp. or exp randomization/
40. double blind procedure/
41. (double-blind or double blind).mp.
42. (single-blind or single blind).mp.
43. (triple-blind or triple blind).mp.
44. random*.mp.
45. interrupted time series.mp.
46. (controlled before and after stud* or controlled before-and-after stud*).mp.
47. controlled before after design.mp.
48. search:.tw.
49. review.pt.
50. systematic review.tw.
51. meta analysis.mp,pt.
52. case series.mp.
53. cost effective:.mp.
54. cost utility:.mp.
55. exp health care costs/
56. (costs and costs analysis).mp.
57. economic evaluation*.mp.
58. ((cost effective* adj1 analys*) or cost minimi?ation analys* or cost benefit analys* or cost utility analys* or cost consequence analys* or finances).mp.
59. or/34–58
60. 21 and 33 and 59

### Search strategy 2: (Cochrane library, HTA, EED, CINAHL, ISI Web of Science, TRIP)

(Insect sting or insect bite or bee sting or bumble bee sting or wasp sting or paper wasp sting or ant sting or fire ant sting or insect allergy or venom allergy or insect venom allergy or bee sting allergy or wasp sting allergy or fire ant allergy or hypersensitivity or immediate type hypersensitivity or delayed hypersensitivity or allergic reaction or severe allergic reaction or anaphylaxis or anaphylactic shock)

AND

(Immunologic, desensiti* or immunotherapy or venom immunotherapy or specific immunotherapy for hymenoptera venom or subcutaneous immunotherapy or sublingual immunotherapy or intradermal immunotherapy or intralymphatic immunotherapy or specific immunotherapy)

AND

(Intervention stud* or experimental stud* or trial or clinical trial* or controlled clinical trial or randomi* controlled trial or quasi-ranomi* controlled trial or random allocation or single blind method or double blind method or triple blind method or random* or interrupted time series or controlled before and after stud* or systematic review or meta-analysis or case series or economic evaluation* or cost effective* analys* or cost minimi?ation analys* or cost benefit analys* or cost utility analys* or cost consequence analys* or finances).
