# Erratum to: Allergen immunotherapy for insect venom allergy: protocol for a systematic review

**DOI:** 10.1186/s13601-017-0165-8

**Published:** 2017-09-15

**Authors:** Sangeeta Dhami, Ulugbek Nurmatov, Eva-Maria Varga, Gunter Sturm, Antonella Muraro, Cezmi A. Akdis, Darío Antolín-Amérigo, M. Beatrice Bilò, Danijela Bokanovic, Moises A. Calderon, Ewa Cichocka-Jarosz, Joanna N. G. Oude Elberink, Radoslaw Gawlik, Thilo Jakob, Mitja Kosnik, Joanna Lange, Ervin Mingomataj, Dimitris I. Mitsias, Holger Mosbech, Oliver Pfaar, Constantinos Pitsios, Valerio Pravettoni, Graham Roberts, Franziska Ruëff, Betül Ayşe Sin, Aziz Sheikh

**Affiliations:** 1 Evidence-Based Health Care Ltd, Edinburgh, UK; 2 Systematic Review at Decision Resources Group, Abacus International, Bicester, UK; 3 Respiratory and Allergic Disease Division, Department of Pediatric and Adolescent Medicine, Medical University of Graz, Graz, Austria; 4 Department of Dermatology and Venerology, Medical University of Graz and Outpatient Allergy Clinic Reumannplatz, Vienna, Austria; 5 Department of Women and Child Health, Food Allergy Referral Centre Veneto Region, Padua General University Hospital, Padua, Italy; 6 Swiss Institute of Allergy and Asthma Research (SIAF), University of Zurich, Zurich, Switzerland; 7 Servicio de Enfermedades del Sistema Inmune-Alergia, Departamento de Medicina y Especialidades Médicas, Hospital Universitario Príncipe de Asturias, Madrid, Spain; 8 Universidad de Alcalá, Madrid, Spain; 9 Allergy Unit, Department of Internal Medicine, University Hospital of Ancona, Ancona, Italy; 10 Department of Dermatology and Venerology, Medical University of Graz, Graz, Austria; 11 Section of Allergy and Clinical Immunology, Imperial College London, London, UK; 12 National Heart and Lung Institute, Royal Brompton Hospital, London, UK; 13 Department of Pediatrics, Jagiellonian University Medical College, Kraków, Poland; 14 Department of Allergology Internal Medicine, University Medical Hospital Groningen, University of Groningen, Groningen, The Netherlands; 15 Department of Internal Medicine, Allergy and Clinical Immunology, Medical University of Silesia, Katowice, Poland; 16 Department of Dermatology and Allergology, University Medical Center Gießen and Marburg (UKGM), Justus Liebig University Gießen, Gießen, Germany; 17 Medical Faculty Ljubljana, University Clinic of Respiratory and Allergic Diseases Golnik, Golnik, Slovenia; 18 Department of Pediatric Pneumonology and Allergy, Medical University of Warsaw, Warsaw, Poland; 19 Department of Allergollogy and Clinical Immunology, Mother Theresa School of Medicine, Tirana, Albania; 20 Department of Paraclinical Disciplines, Faculty of Technical-Medical Sciences, Medicine University of Tirana, Tirana, Albania; 21 Department of Allergy and Clinical Immunology, 2nd Pediatric Clinic, University of Athens, Athens, Greece; 22 Allergy Clinic, Copenhagen University Hospital Gentofte, Gentofte, Denmark; 23 Department of Otorhinolaryngology, Head and Neck Surgery, Center for Rhinology and Allergology, University Hospital, Mannheim, Wiesbaden, Germany; 24 Department of Nutrition and Dietetics, Harokopio University, Athens, Greece; 25 Allergy Outpatient Clinic, Apollonio Hospital, Nicosia, Cyprus; 26 UOC Clinical Allergy and Immunology, IRCCS Foundation Ca’ Granda Ospedale Maggiore Policlinico, Milan, Italy; 27 NIHR Respiratory Biomedical Research Unit, The David Hide Asthma and Allergy Research Centre, St Mary’s Hospital, Newport Isle of Wight, University Hospital Southampton NHS Foundation Trust, Southampton, UK; 28 Faculty of Medicine, University of Southampton, Southampton, UK; 29 Klinik und Poliklinik für Dermatologie und Allergologie, Ludwig-Maximilians-Universität, Munich, Germany; 30 Division of Immunology and Allergy, Department of Pulmonary Diseases, Faculty of Medicine, Ankara University, Ankara, Turkey; 31 Allergy and Respiratory Research Group, The University of Edinburgh, Edinburgh, UK

## Erratum to: Clin Transl Allergy (2016) 6:6 DOI 10.1186/s13601-016-0095-x

Unfortunately this article [1] was published with an error in the Funding section. The BM4SIT project is not acknowledged. This section should be corrected to the below:

**Funding**

EAACI and the BM4SIT project (Grant Number 601763) in the European Union’s Seventh Framework Programme FP7.
